# Supplementary material for: Differential Expression and Diagnostic Value of MUC5AC Glycoforms in Pancreatic Ductal Adenocarcinoma
Source: Cancers (Basel). 2023 Oct 2;15(19):4832. doi: 10.3390/cancers15194832 (PMC10571547; doi:10.3390/cancers15194832)
Supplement: Supplementary file 1 [file cancers-15-04832-s001.zip › cancers-2629714-supplementary.pdf]

DDifferential Expression and Diagnostic Value of MUC5AC Glycoforms in Pancreatic Ductal Adenocarcinoma

Supplementary Tables

Supplementary Table S1: Location-wise expression pattern of mature MUC5AC in association with immature isoform in the primary tumors.

Location of MM expression	Stage I/II/III	IM-positive	IM-negative	Total
Ap	3/8/0	9	2	11
Cy	5/10/0	14	1	15
Ec	3/6/0	9	0	9
Ap + Cy	2/6/0	7	1	8
Ap + EC	2/4/0	6	0	6
Cy+ Ec	2/6/0	8	0	8

MM - mature MUC5AC, IM – immature MUC5AC, Ap – apical expression, Cy- cytoplasmic expression, Ec – extracellular expression.

Supplementary Table S2: Stage-wise breakdown of MUC5AC glycoform expression in primary tumors of the tested tissue microarray

	N	MM ± IM	MM-positive	MM-only	IM-only	MM + IM	IM-positive	All negative
Stage I	14	8	6	0	2	6	8	6
Stage II	22	19	12	2	7	10	17	3
Stage III	2	0	0	0	0	0	0	2
Total	38	27	18	2	9	16	25	11
Stage I-II vs III-IV (%) (p-value)	NS	65 vs 35 (p=0.02)	NS	NS	NS	NS	60% vs 40% (p=0.006)	NS
Stage I vs II vs III (%)	NS	57 vs 86 vs 0 (p=0.01)	NS	NS	NS	NS	NS	NS
Stage I vs. II vs. III vs. IV (%)	NS	57 vs 86 vs 0 vs 25 (p=0.04)	NS	NS	NS	NS	57 vs 77 vs 0 vs 25 (p=0.02)	NS
G 1-2 differentiation	22	18	13	2	3	11	16	4
G3, undifferentiated and adenosquamous	16	9	5	0	4	5	8	7
N0 disease	30	20	13	1	7	12	19	10
N1 disease	8	7	5	1	4	2	6	1
T-T2	18	12	9	1	3	8	11	6
T3-T4 disease	20	14	9	1	7	8	14	5

MM - mature MUC5AC, IM – immature MUC5AC, G- grade. NS- not significantly different ($p > 0.05$)
